# Nicotinamide Prevents Diabetic Brain Inflammation via NAD+-Dependent Deacetylation Mechanisms

**DOI:** 10.3390/nu15143083

**Published:** 2023-07-09

**Authors:** Jeimy Katherine Torres-Méndez, Julia Niño-Narvión, Patricia Martinez-Santos, Elena María Goretti Diarte-Añazco, Karen Alejandra Méndez-Lara, Tania Vázquez del Olmo, Noemi Rotllan, Maria Teresa Julián, Núria Alonso, Didac Mauricio, Mercedes Camacho, Juan Pablo Muñoz, Joana Rossell, Josep Julve

**Affiliations:** 1 Institut d’Investigació Biomèdica Sant Pau (IIB Sant Pau), 08041 Barcelona, Spain; jeimykatherine.torres@estudiants.urv.cat (J.K.T.-M.); julianng99@gmail.com (J.N.-N.); pat.martinez@gmail.com (P.M.-S.); goretti.diarte@gmail.com (E.M.G.D.-A.); kmendez1101@gmail.com (K.A.M.-L.); nrotllan@santpau.cat (N.R.); didacmauricio@gmail.com (D.M.); mcamacho@santpau.cat (M.C.); pmunozn@santpau.cat (J.P.M.); 2 Departamento de Bioquímica y Biología Molecular B e Inmunología, Facultad de Medicina, Universidad de Murcia (UMU), 30120 Murcia, Spain; 3 Department of Pathology, Hospital de la Santa Creu i Sant Pau, 08041 Barcelona, Spain; tvazquez@santpau.cat; 4 CIBER de Diabetes y Enfermedades Metabólicas Asociadas, Instituto de Salud Carlos III, 28029 Madrid, Spain; mtjulian.germanstrias@gencat.cat (M.T.J.); nalonso32416@yahoo.es (N.A.); 5 Department of Endocrinology & Nutrition, Hospital Universitari Germans Trias i Pujol, 08916 Badalona, Spain; 6 Department of Endocrinology & Nutrition, Hospital de la Santa Creu i Sant Pau, 08041 Barcelona, Spain; 7 Faculty of Medicine, University of Vic/Central University of Catalonia (UVIC/UCC), 08500 Vic, Spain

**Keywords:** type 1 diabetes mellitus, macrophage, sirtuin, activated microglia, neurological disease, neuropathy, neuroinflammation

## Abstract

This study investigated the effect of nicotinamide (NAM) supplementation on the development of brain inflammation and microglial activation in a mouse model of type 1 diabetes mellitus. C57BL/6J male mice, which were made diabetic with five consecutive, low-dose (55 mg/kg i.p.) streptozotocin (STZ) injections. Diabetic mice were randomly distributed in different experimental groups and challenged to different doses of NAM (untreated, NAM low-dose, LD, 0.1%; NAM high-dose, HD, 0.25%) for 25 days. A control, non-diabetic group of mice was used as a reference. The NAD+ content was increased in the brains of NAM-treated mice compared with untreated diabetic mice (NAM LD: 3-fold; NAM HD: 3-fold, *p*-value < 0.05). Immunohistochemical staining revealed that markers of inflammation (TNFα: NAM LD: −35%; NAM HD: −46%; *p*-value < 0.05) and microglial activation (IBA-1: NAM LD: −29%; NAM HD: −50%; *p*-value < 0.05; BDKRB1: NAM LD: −36%; NAM HD: −37%; *p*-value < 0.05) in brains from NAM-treated diabetic mice were significantly decreased compared with non-treated T1D mice. This finding was accompanied by a concomitant alleviation of nuclear NFκB (p65) signaling in treated diabetic mice (NFκB (p65): NAM LD: −38%; NAM HD: −53%, *p*-value < 0.05). Notably, the acetylated form of the nuclear NFκB (p65) was significantly decreased in the brains of NAM-treated, diabetic mice (NAM LD: −48%; NAM HD: −63%, *p*-value < 0.05) and inversely correlated with NAD+ content (r = −0.50, *p*-value = 0.03), suggesting increased activity of NAD+-dependent deacetylases in the brains of treated mice. Thus, dietary NAM supplementation in diabetic T1D mice prevented brain inflammation via NAD+-dependent deacetylation mechanisms, suggesting an increased action of sirtuin signaling.

## 1. Introduction

Diabetes is one of the leading causes of neuropathy [1]. Diabetic neuropathy is characterized by progressive nerve damage and dysfunction that may result in enhanced pain and other disabling sensorial manifestations [1]. Although a traditional view of this condition can be defined as painful nerve damage mainly limited to peripheral areas, affecting distal sensory nerves in the lower extremities, new research suggests that may be also harmful to the diabetic brain [2,3].

The pathogenesis of diabetic brain injury during long-term diabetes is complex, but it has been linked to both vascular and non-vascular mechanisms associated with chronic hyperglycemic states [3]. A deficient cerebral blood supply and metabolic derangements related to chronic hyperglycemia and altered insulin signaling may contribute to brain damage, inflammation, and subsequent neurodegeneration [2,3,4].

The brain consumes large amounts of oxygen and ATP predominantly from glucose to sustain its normal function and thus this tissue is highly prone to enhanced oxidative stress [5]. Under hyperglycemic states, glucose metabolism shifts to the polyol and hexosamine pathways [6], thereby exacerbating oxidative stress. In turn, the advanced glycation end-product (AGE) production is also enhanced, which leads to increased oxidative stress and production and systemic and local release of proinflammatory molecules via interaction with AGE receptors [6].

Diabetes induces intracellular metabolic stress, thus ensuing cellular inflammation and microglia activation, which profoundly impairs brain physiology and function [5]. Within the diabetic brain, macrophage-resident cell (microglia) activation further leads to subsequent cytokine and chemokine production, which perpetuates and amplifies the local inflammatory response, thereby contributing to nervous system dysfunction [5]. In this context, nuclear factor kappa B (NFκB), a master regulator of a large array of genes involved in different processes of inflammation and immune response [7], is activated by hyperglycemia [8]. NFκB dimers are present in a latent inactive form in the cytoplasm bound to IκB inhibitory proteins. Under inflammatory conditions, IκB kinase complex phosphorylates IκB, which triggers its subsequent ubiquitin-dependent degradation in the proteasome and nuclear translocation of active canonical NFκB members, predominantly the p50/RelA(p65) and p50/c-Rel dimers [9]. Upon activation, NFκB induces the transcription of molecular targets involved in different cellular processes, including inflammation [10].

NAD+ depletion profoundly shapes the clinical outcome of diabetic brain inflammation [11]. As such, changes in NAD+ metabolism observed in diabetic subjects may reflect endogenous declines of NAD+ precursors (including NAM and their main related derivatives). Among the potential mechanisms underlying such endogenous NAD+ depletion may be tissue-specific and possibly underlie the different clinical complications of diabetes, including brain deterioration [12]. In this regard, excess consumption of NAD+ equivalents due to alternatively-induced pathways of glucose metabolism in brains exposed to persistent hyperglycemic states may contribute to concomitant NAD+ reductions, mainly due to the induction of the poly (ADP-ribose) polymerase [13,14].

NAD+-dependent histone deacetylases, also known as sirtuins (SIRT), are a class of NAD+-consuming enzyme which is involved in the deacetylation of different signaling proteins [11]. Particularly, SIRT1 is able to inhibit NFκB action by specifically deacetylating the p65 subunit, thereby suppressing inflammation [15]. Recent research suggests that sirtuin activity can be enhanced by the administration of NAD+ precursors, i.e., nicotinamide mononucleotide and nicotinamide riboside, to alleviate neuroinflammation in the central nervous system [16,17,18,19,20].

NAD+ replenishment is emerging as a potential therapy to combat the development of neurological manifestations in diabetic subjects [21]. Nicotinamide (NAM), the amide form of vitamin B3, is also a NAD+ precursor [22]. NAM has long been associated with neuronal development and function in the central nervous system and is involved in both neuronal death and neuroprotection [12]. NAM may also favorably influence cerebral inflammation [23,24,25]; however, the impact on deacetylase enzymatic activity of this or any other NAD+ precursors on NFκB signaling and microglia activation has not been addressed in any of these studies. In this regard, we hypothesized that NAM may protect against brain inflammation and activated microglia via NAD+-dependent NFκB deacetylation mechanisms. Thus, such NAM-mediated anti-inflammatory mechanism was investigated in the brains of mouse models of diabetes mellitus type 1 induced with streptozotocin (STZ).

## 2. Materials and Methods

### 2.1. Mice and Treatments

All experimental procedures were in accordance with the Directives of the European Union Council (2010/63/UE) and of the Spanish Government (RD 53/2013) for the use of animals in research. All efforts were made to minimize animal suffering and to reduce the number of animals used. All animal protocols were reviewed and approved by the local Animal Care and Use Committee of our institution (Institut de Recerca de l’Hospital de la Santa Creu i Sant Pau; Procedure Nº 10434), and the methods were conducted in accordance with the approved guidelines. This study on the effect of NAM administration was performed in male C57BL/6NCrl mice obtained from Charles River Laboratories International, Inc. (strain code 027; Bar Harbor, ME). All mice were adult (8 weeks old) at the beginning of the study and were housed in conditions of a temperature-controlled environment (20 °C) with 66% humidity and a 12-h light/dark cycle. Animals had free access to food and water. Food intake and water consumption were monitored in individually housed mice for 48 h. Mice were exsanguinated directly from the heart at the end of the procedure, and blood was placed into tubes containing EDTA as an anticoagulant. Plasma was stored at −70 °C before analysis. The brains were removed from the skull after euthanizing the animals by cervical dislocation, frozen in liquid nitrogen, and stored at −70 °C or fixed in 10% neutral buffered formalin solution (cat# HT501128, Merck KGaA, Darmstadt, Germany), as appropriate. The two hemispheres were separated along the cerebral longitudinal (sagittal) fissure. The hemispheres were randomly sampled and used for analyses.

### 2.2. Assessment of NAM on Diabetic Brain Inflammation Induced with STZ

**Figure 1:**
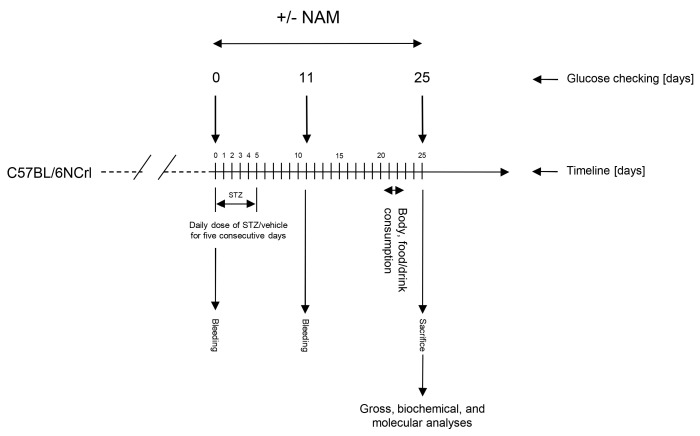
Schematic representation of the experimental design and mice treatments. Gross, biochemical, histological, and molecular analyses were carried out at the end of the study. C57BL/6 mice fed a regular chow diet were used. Diabetes was induced by the intraperitoneal administration of five consecutive daily injections of 55 mg/kg streptozotocin (STZ) (Sigma-Aldrich, St. Louis, MO, USA) freshly prepared in citrate buffer (0.1 M, pH 4.5). Diabetes was assessed by measuring blood glucose levels using an AccuCheck glucometer (Roche Diagnostics). After injection, STZ-administered animals were randomly distributed into three groups (n = 6 in each group) according to the oral dose of NAM dissolved in drinking water from the beginning until day 25 after STZ injection. An untreated group of mice made diabetic was used to compare the effect of NAM manipulation. A group of untreated, non-diabetic mice was used as a reference to compare the impact of diabetes. Abbreviations used: NAM LD, low-dose, NAM-treated mice; NAM HD, high-dose, NAM-treated mice; STZ, streptozotocin.

Diabetes was induced by the intraperitoneal administration of five consecutive daily injections of 55 mg/kg STZ (cat# S0130, Sigma-Aldrich, St. Louis, MO, USA) freshly prepared in citrate buffer (0.1 M, pH 4.5) (Figure 1). Diabetes was assessed by measuring blood glucose levels using an AccuCheck glucometer (Roche Diagnostics). After injection, STZ-administered animals were randomly distributed into three groups (n = 6 in each group) according to the oral dose of NAM dissolved in drinking water (i.e., 0.1% and 0.25%) from the beginning until day 25 after STZ injection. The diabetic group of mice that did not receive treatment was defined as untreated, diabetic mice. A non-diabetic group receiving non-supplemented water was used as a control. The doses and time points of NAM chosen were selected from preliminary experiments shown to be effective in producing inflammation relief in other target tissues [26,27] as well as from our previous pilot studies performed in our mouse model of STZ-induced diabetic brain inflammation. Daily NAM consumption was estimated in individually-housed mice only in the treated groups. NAM intake was calculated by multiplying the individual daily volume of water consumption by the concentration (in %) of dissolved NAM, as appropriate. NAM intake was expressed as the amount of daily uptake of NAM per kg of body weight.

### 2.3. Laboratory Analysis

Most plasma chemicals, including cholesterol (cat# 3039773190); triglycerides (cat# 20767107322); glucose (cat#4404483190); alanine aminotransferase (ALT, cat# 20764949322), and aspartate aminotransferase (AST, cat# 20764949322) activities were determined using commercial kits adapted to a COBAS c501 autoanalyzer (Roche Diagnostics, Madrid, Spain). Circulating triglycerides were corrected for the free glycerol present in plasma (cat# F6428-40ML; Sigma-Aldrich St. Louis, MO). Plasma concentrations of cytokines (IL-10, IL-6, IL-4, and TNFα) were analyzed using Luminex xMAP^®^ technology (Millipore Corporation, Billerica, MA, USA). Plasma insulin was measured using the Ultra-Sensitive Mouse Insulin Elisa Kit (cat# 90080, Crystal Chem Inc., Downers Grove, IL, USA). NAD+ levels were determined in brain tissue using an NAD/NADH colorimetric determination kit (cat# ab65348 Abcam) according to the manufacturer’s instructions. Briefly, 50 mg was homogenized using a polytron with 400 µL of extraction buffer. Samples were filtered through a 10 kD Spin Column (cat# ab93349) before performing the assay in the collected filtrates. The absorbance was eventually read at a wavelength of 450 nm in a Beckman spectrophotometer AD340 (Beckman). The protein concentration of the supernatant was evaluated by the bicinchoninic acid technique (BCA protein assay kit; cat# 23227, Thermo Scientific, Waltham, MA, USA).

### 2.4. Immunohistochemistry Analysis

Random sagittal segments of brain hemispheres were fixed with a 10% neutral buffered formalin solution (cat# HT501128, Merck KGaA, Darmstadt, Germany). Seven-micrometer sections of paraffin-embedded tissue samples were incubated with rabbit polyclonal antibodies against TNFα (cat# GTX110520, diluted 1:200, *v*:*v*) from GeneTex, Ionized Calcium-Binding Adapter Molecule 1 (IBA-1) (cat# 019-19741, diluted 1:2000, *v*:*v*) from FUJIFILM Wako Pure Chemical Corporation, B1 Bradykinin Receptor (BDKRB1)-1 (cat# bs-8675R, diluted 1:500, *v*:*v*) from Bioss, NFκB (p65) (clone D14E12) (cat# 8242, diluted 1:1000, *v*:*v*) from Cell Signaling, NFκB (p65) (acetyl K310) (cat# ab19870, diluted 1:300, *v*:*v*) from Abcam, and F4/80 (clone D4C8V) (cat# 30325, diluted 1:200, *v*:*v*) from Cell Signaling for 30 min and stained with diaminobenzidine (DAB) in a Dako Omnis instrument using the EnVision FLEX Target Retrieval Solution LOW pH (Dako Omnis, cat# GV805), EnVision FLEX Rabbit LINKER, (Dako Omnis, cat# GV809), and Dako EnVision+System-HRP-DAB-kit, according to the manufacturer’s protocol. Negative controls using the rabbit secondary antibodies further validated the results of immunohistochemical staining in brain tissue (Figure S1). Slides were then dehydrated and coverslipped and images were obtained using a Pannoramic SCAN II and Pannoramic Scanner 3.0.3. RTM software. Slides were visualized using the Slide Viewer software version 2.5. We performed immunostaining analysis of brain slices of 4–5 mice/group using the image analysis protocol described by Crowe and Yue [28]. Cerebral cortex images under similar magnification (scale bar 100 µm adjusted for pixel) of 10 randomly selected fields were used for quantification. Images were quantified using ImageJ-Fiji software. Briefly, diaminobenzidine and hematoxylin IHC stained images were collected using Pannoramic SCAN and exported to ImageJ (NIH) software. Image were deconvoluted using ImageJ “Color deconvolution tool” and then to generate binary images, each individual images were thresholding. Images were quantified using Analyze “Mean grey value” ImageJ tool and data were processed using GraphPad Prism software (GPAD, version 5.0, San Diego, CA, USA).

### 2.5. Western Blotting

For Western Blotting, 100 mg of brain tissue was homogenized in RIPA (150 mM NaCl, 10 mM Tris (pH 7.2), 0.1% SDS, 1% Triton X-100, 1% deoxycholate, 5 mM EDTA, supplemented with protease inhibitor mixture and phosphatase inhibitor) and centrifuged at 10,000× *g* for 20 min at 4 °C. Then, 20–40 µg of proteins from total homogenates were resolved in 12% acrylamide gels for SDS-PAGE and transferred to Immobilon-FL membranes (Millipore, Burlington, MA, USA). NFκB (p65) (acetyl K310) (cat# ab19870) were diluted 1:1000 (*v*:*v*) in PBS with 1% BSA and 0.1% Tween 20. Mouse anti-GAPDH used at 1:20,000 (*v*:*v*) (clone 6C5 #MAB374 from Sigma-Aldrich) or Revert™ 700 Total Protein Stain for Western Blot Normalization (LI-COR) was used as a loading control. Secondary antibodies were diluted 1:20,000 (*v*:*v*) in 1% BSA and 0.1% Tween 20. Proteins were detected using the Odyssey Infrared Image System (LI-COR).

### 2.6. Quantitative Real-Time RT-PCR Analysis

Total RNA was isolated from brain tissue using the TRIzol RNA isolation method (Ambion, cat# 15596018; Life Technologies, Carlsbad, CA, USA). Total RNA samples were then repurified (RNeasy mini kit Plus, cat# 74134; Qiagen, CA, USA). Total mRNA (1 μg) was reverse-transcribed with Oligo(dT)_15_ using M-MLV Reverse Transcriptase, RNase H Minus, and Point Mutant (Promega Corporation, MD, USA) to generate cDNA. Predesigned validated primers (Assays-on-Demand; Life Technologies) were used with specific TaqMan probes. Specific mouse Taqman probes (Life Technologies) were used for *Aif1* (Mm00479862_g1), *Bdkrb1* (Mm04207315_s1), and the reference genes *Actb* (Mm99999903_g1) and *Gapdh* (Mm99999905_m1). Real-time PCR assays were performed on a C1000 Thermal Cycler coupled to a CFX96 Real-Time System (Bio-Rad Laboratories SA, Life Science Group, Madrid, Spain). All analyses were performed in duplicate. The relative mRNA expression levels were calculated by the ΔΔCt method.

### 2.7. Statistical Analysis

The data were expressed as the mean ± standard error of the mean (SEM). The effects of diabetes or NAM treatment on gross and plasma chemical parameters, or the relative levels of gene/protein expression were determined using parametric one-way ANOVA followed by Tukey’s posttest. The relationship between variables was tested with Pearson’s correlation. Statistical analyses were performed using GraphPad Prism software (GPAD, version 5.0, San Diego, CA, USA). A *p*-value < 0.05 was considered statistically significant.

## 3. Results

### 3.1. NAM Effects on Gross and Biochemical Parameters of Diabetic Mice

The impact of NAM on gross and basic biochemical data of mice with type 1 diabetes mellitus (T1D) is shown in Table 1. Food intake and water consumption were significantly increased in all groups of diabetic mice. The calculated NAM intake was increasingly higher in the diabetic mice being proportional to the administered dose. The NAM intake by diabetic mice treated with the NAM HD almost doubled that treated with the NAM LD. The body weight was reduced in diabetic mice; the body weight did not differ in diabetic mice treated with NAM. Neither the liver, kidneys, nor brain weights did differ among groups regardless, of diabetes or treatment.

Diabetic mice displayed glucose elevations (>3-fold, *p*-value < 0.05) compared with non-diabetic mice. Hyperglycemia was not influenced by the treatment with NAM. As expected, insulin concentrations were significantly lower in diabetic mice compared with non-diabetic mice. NAM administration did not influence serum insulin concentrations, as shown by the absence of differences among diabetic groups. The treatment with NAM did not affect the circulating levels of hepatic transaminases. Plasma concentrations of total cholesterol were similar in all groups of mice; however, total triglycerides were higher in diabetic mice compared to those shown by non-diabetic mice. Noteworthy, the serum concentration of this lipid was significantly reduced only in diabetic mice receiving the NAM LD treatment.

**Figure 2:**
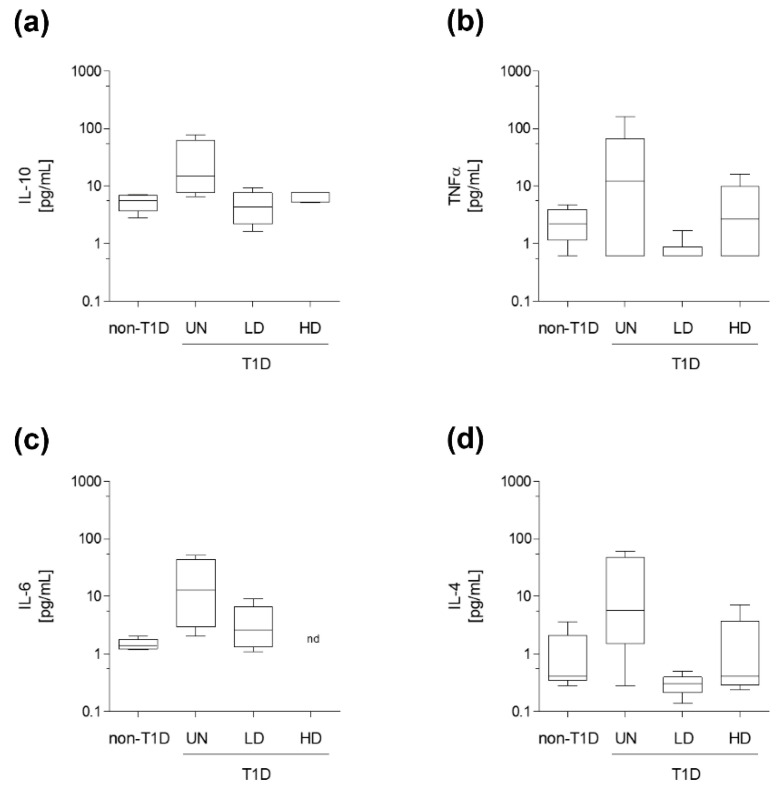
Circulating cytokines. (**a**) Plasma IL-10. (**b**) Plasma IL-6. (**c**) Plasma IL-4. (**d**) Plasma TNFα. Data are expressed as the mean (standard deviation) of 5–6 mice/group. Statistically significant differences among groups for each variable were determined using a parametric ANOVA test followed by Tukey’s posttest. Differences were considered significant when *p*-value < 0.05. Abbreviations used: NAM LD, low-dose, NAM-treated, diabetic mice; NAM HD, high-dose, NAM-treated, diabetic mice; non-T1D, group of mice without T1D; T1D, group of mice with T1D; UN, untreated, diabetic mice; nd, not determined.

Among the different cytokines analyzed (Figure 2), neither the levels of pro-inflammatory TNFα, IL-6, nor the anti-inflammatory IL-10, IL-4 differed among groups.

### 3.2. NAM Attenuated NFκB Signaling and Microglial Activation in Diabetic Mice

**Figure 3:**
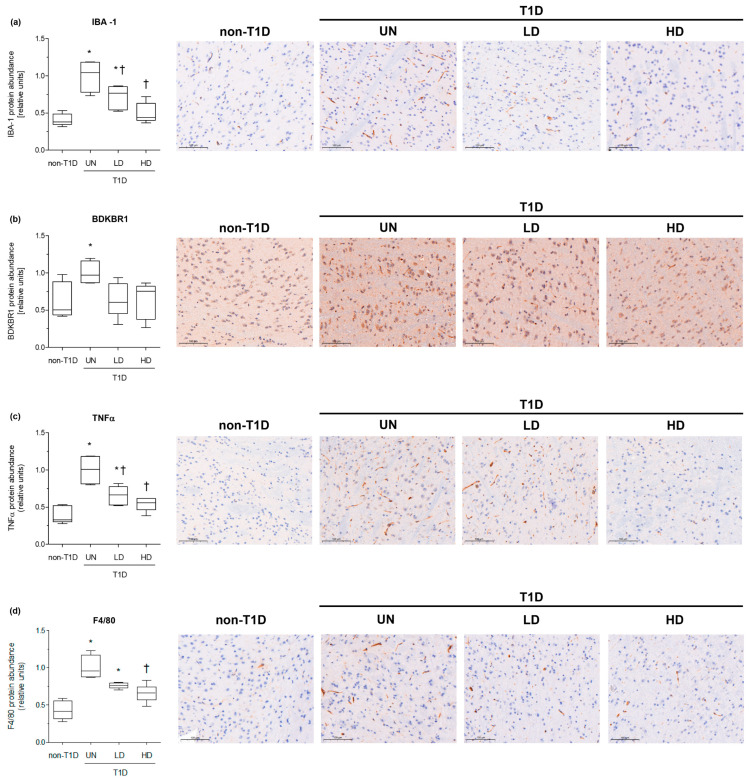
Effect of NAM on the relative levels of brain biomarkers of inflammation and microglial activation in STZ-induced diabetic mice. (**a**) Relative protein abundance of brain IBA-1 and representative images of specific IHC in each condition. (**b**) Relative protein abundance of brain BDKRB1 and representative images of specific IHC in each condition. (**c**) Relative protein abundance of brain TNFα and representative images of specific IHC in each condition. (**d**) Relative protein abundance of brain F4/80 and representative images of specific IHC in each condition. The scale bar shown in the images represents 100 µm. Data are expressed as the mean (standard deviation) of 4–5 mice/group. Images of 10 randomly selected fields were used for quantification. Statistically significant differences among groups for each variable were determined using a parametric ANOVA test followed by Tukey’s posttest. Differences were considered significant when *p*-value < 0.05. Specifically, * *p*-value < 0.05 vs. non-diabetic group. † *p*-value < 0.05 vs. diabetic group. Abbreviations used: LD, low-dose, NAM-treated, diabetic mice; HD, high-dose, NAM-treated, diabetic mice; non-T1D, group of mice without T1D; T1D, group of mice with T1D; UN, untreated, diabetic mice.

Next, we investigated whether NAM treatment altered the phenotype of microglia (Figure 3). The relative protein abundance of microglial markers IBA-1 and BDKRB1 receptor was significantly elevated in the brains of diabetic mice. NAM-treated mice exhibited significant reductions of these molecular targets of microglial activation (Figure 3, panels a–c). In line with this, the relative gene expression levels of both markers (*Aif1* and *Bdkrb1*) of activated microglia were overall decreased in NAM-treated mice compared with untreated diabetic mice (Figure S2). The content of TNFα was also raised in diabetic mice compared with non-diabetic mice and was significantly reduced in the diabetic mice groups treated with NAM (Figure 3). F4/80, an unspecific marker of macrophages, was also reduced in the brains of NAM-treated, diabetic mice (34%, *p*-value < 0.05) compared with untreated diabetic mice (Figure 3, panel d), also suggesting reduced macrophage recruitment in brains from treated diabetic mice.

**Figure 4:**
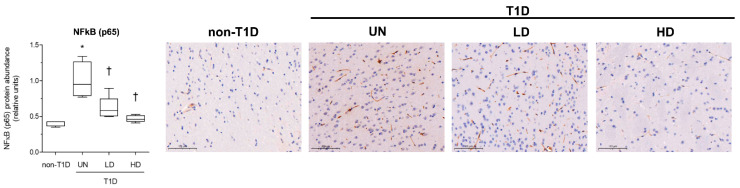
Analysis of the effect of NAM on the relative levels of total NFκB (p65) in brains of STZ-induced diabetic mice. Relative protein abundance of brain NFκB (p65) and representative images of brain IHC in each condition. Data are expressed as the mean (standard deviation) of 4–5 mice/group. Images of 10 randomly selected fields were used for quantification. The scale bar shown in the images represents 100 µm. Statistically significant differences among groups for each variable were determined using a parametric ANOVA test followed by Tukey’s posttest. Differences were considered significant when *p*-value < 0.05. Specifically, * *p*-value < 0.05 vs. non-diabetic group; † *p*-value < 0.05 vs. diabetic group. The relationship between parameters was tested using a parametric Pearson’s correlation test. Mice of all groups were considered for analysis. Abbreviations used: acetylated form of NFκB (p65), acetyl-NFκB (p65); LD, low-dose, NAM-treated, diabetic mice; HD, high-dose, NAM-treated, diabetic mice; non-T1D, group of mice without T1D; T1D, group of mice with T1D; UN, untreated, diabetic mice.

The protein abundance of the phosphorylated form of the nuclear, transcriptionally active form NFκB (p65) was concomitantly raised in untreated diabetic mice and downregulated in the NAM-treated mice, thereby suggesting that this signaling was significantly inhibited (Figure 4). Collectively, our findings suggested that NAM administration negatively influenced brain inflammation and concomitant microglial activation in NAM-treated, diabetic mice.

### 3.3. NAM Enhanced NAD+-Dependent Deacetylation of NFκB to Ameliorate Brain Inflammation of Diabetic Mice

**Figure 5:**
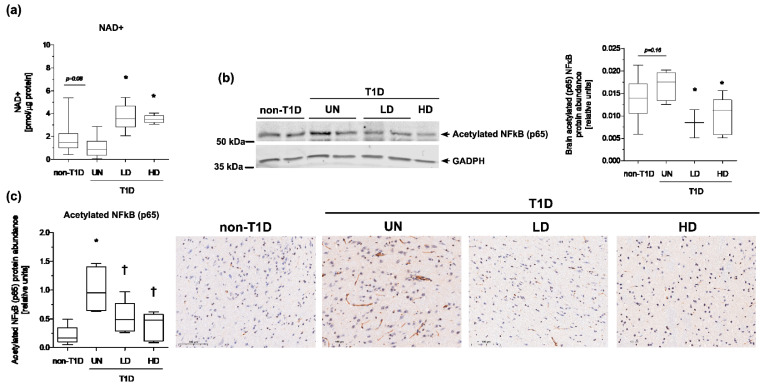
Effect of NAM on the brain NAD+ content and acetylated form of NFκB (p65) in brains of STZ-induced diabetic mice. (**a**) Brain NAD+ content. (**b**) Relative protein abundance of the acetylated NFκB (p65) obtained by western blot analysis and representative images of brain acetylated NFκB (p65) in each condition. (**c**) Relative protein abundance of the acetylated NFκB (p65) obtained by IHC analysis and representative images of brain acetylated NFκB (p65) in each condition. Abbreviations used: NAD+, oxidized form of nicotinamide adenine dinucleotide; NAM LD, low-dose, NAM-treated, diabetic mice; NAM HD, high-dose, NAM-treated, diabetic mice; non-T1D, group of mice without T1D; T1D, group of mice with T1D. Data are expressed as the mean (standard deviation) of 4–5 mice/group. Images of 10 randomly selected fields were used for quantification. The scale bar shown in the images represents 100 µm. Statistically significant differences among groups for each variable were determined using a parametric ANOVA test followed by Tukey’s posttest. Differences were considered significant when *p*-value < 0.05. Specifically, * *p*-value < 0.05 vs. non-diabetic group; † *p*-value < 0.05 vs. diabetic group.

NAM can freely cross the blood-brain barrier in both directions [29] and is considered a NAD+ precursor [29]. Therefore, to further explore the specific mechanism of NAM, we first assessed the content of NAD+ in the brain tissue of diabetic mice and the impact of NAM treatments (Table 1, Figure 5a). Although the reduction of NAD+ content in the brains of our diabetic mice was just marginal (*p*-value = 0.08) compared with non-diabetic mice, NAM supplementation to diabetic mice significantly increased brain NAD+ stores compared with the untreated, diabetic counterparts. (Table 1, Figure 5a).

NAD+ is a coenzyme for multiple signaling reactions [30], including those catalyzed by a class of protein deacetylases, collectively known as sirtuins, which, when elevated, can deacetylate NFκB and hence inhibit its activity and inflammatory signaling. Therefore, we directly studied the relative brain content of the nuclear acetylated NFκB (p65) in the brains of mice. Our results showed that the relative content of acetylated NFκB (p65) was significantly decreased in the brains of NAM-treated, diabetic mice (Figure 5b,c and Figure S3).

**Figure 6:**
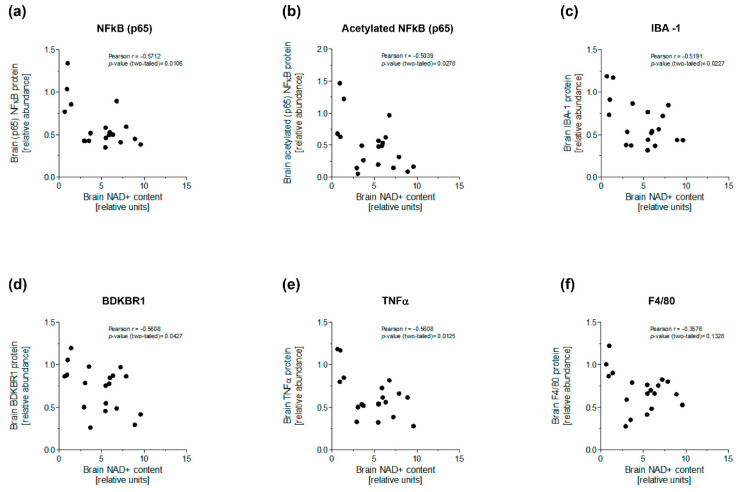
Relationship between relative NAD+ content and biomarkers of inflammation and microglial activation in brains from diabetic mice. (**a**) Correlation between relative levels of brain NAD+ content and IHC protein abundance of brain NFκB (p65). (**b**) Correlation between relative levels of brain NAD+ content and IHC protein abundance of brain acetyl-NFκB (p65). (**c**) Correlation between relative levels of brain NAD+ content and IHC protein abundance of brain IBA-1. (**d**) Correlation between relative levels of brain NAD+ content and IHC protein abundance of brain BDKRB-1. (**e**) Correlation between relative levels of brain NAD+ content and IHC protein abundance of brain TNFα. (**f**) Correlation between relative levels of brain NAD+ content and IHC protein abundance of brain F4/80. The relationship between parameters was tested using a parametric Pearson’s correlation test. The relative levels of brain NAD+ were calculated taking as 1 the mean of individual values for this parameter in untreated diabetic mice. Mice of all groups were considered for analysis.

Interestingly, the relative content of total NFκB (p65) and acetyl-NFκB (p65) was inversely associated with the relative brain amount of NAD+ (total NFκB (p65): Pearson r = −0.5712, *p*-value = 0.0106; acetyl-NFκB (p65): Pearson r = −0.5039, *p*-value = 0.0278) (Figure 6a,b). Similarly, the relative brain content of activated microglial and inflammatory markers was also inversely correlated with brain NAD+ (IBA-1: Pearson r = −0.5191, *p*-value = 0.0227; BDKBR1: Pearson r = −0.5608, *p*-value = 0.0427; TNFα: Pearson r = −0.5608, *p*-value = 0.0125) (Figure 6c–e). Brain F4/80 was just marginally correlated with brain NAD+ (F4/80: Pearson r = −0.3576, *p*-value = 0.1328) (Figure 6f). These findings suggested that macrophage infiltration and the inflammatory response in brains would be decreased by NAM.

## 4. Discussion

This study investigated the effect of the treatment with the NAD+ precursor NAM on brain inflammation and microglial activation in a mouse model of T1D. A body of evidence suggests that brain inflammation has been strongly linked to nerve tissue NAD+ depletion in different scenarios [19,31,32], including insulin-deficient states [21]. As such, NAD+ replenishment is emerging as a potential therapy to combat the development of neurological manifestations in diabetic subjects [21]. Insomuch as NAD+ acts as an obligatory coenzyme for a class of cellular protein deacetylases collectively known as sirtuins [11], the mechanisms underlying the anti-inflammatory effects by NAD+-increasing approaches may involve direct effects on such enzymes; however, the impact of NAD+-mediated deacetylation on NFκB signaling in brains of diabetic mice have not been addressed in any previous study. In this context, to our knowledge, our findings are the first to show the contribution of NAD+-related deacetylation mechanisms in the attenuation of brain inflammation and microglial activation.

Microglial homeostasis plays a critical role in the regulation of immune response and central nervous system-related diseases [33]. Our data revealed that the relative content of TNFα was significantly decreased in the brains of NAM-treated, diabetic mice. This finding is consistent with previous data [26,34,35] showing an effect of NAM on inhibiting TNFα synthesis and secretion in vitro and in vivo. The infiltration and activation of immune cells is a characteristic of chronic inflammation processes [36,37], thus the relative content of brain F4/80, as a surrogate of macrophage infiltration, also suggested the differential accumulation of macrophages in the brains of diabetic mice.

Another finding in our study was the involvement of favorable reductions in the relative brain content of the acetylated form of (p65) NFκB. This result was linked to a significant amelioration of brain inflammation, as revealed by the lower relative levels of brain TNFα, and also, microglial activation, as shown by the observed reductions in the brain content of IBA-1 and BDKRB1, in NAM-treated, diabetic mice. Microglial cells are the only brain cells to express IBA-1 and are the first responders against inflammatory and pathophysiological stimuli, including AGEs [38]. Recent observations also indicate that cytokines may upregulate BDKRB1 [39]. NAD+-dependent, protein deacetylases determine the cellular acetylation status and have a prominent role in the modulation of immune response and inflammation [40]. Upon diabetes, the activity of this class of enzymes is frequently reduced and generally accompanied by enhanced local inflammation and activated microglia in brain tissue (NMN-microglia-NAD+). Of note, their biological activity is particularly sensitive to fluctuations in the cellular content of NAD+, being at least in part attributed to their higher Michaelis-Menten constant and hence the relatively higher requirement for NAD+ compared with other NAD+-consuming enzymes [41]. Consistently with data from other studies using another NAD+ precursor [42,43,44], the favorable sirtuin-related effects of NAM supplementation on inflammation can be also due to its role as an NAD+ precursor. Indeed, sirtuin-dependent deacetylation could be conceivably reduced in the brains of our diabetic mice, and thus be linked to an impaired anti-inflammatory action [45]. On the contrary, NAD+-dependent deacetylation was increased in diabetic brains, as revealed by the significant decrease in the brain content of the acetylated form of (p65) NFκB and lower molecular signs of inflammation in NAM-treated, diabetic mice. Our data are consistent with previous data whereby NAM-dependent increases of intracellular NAD+ concentration negatively influenced TNFα production by activated immune cells at a post-transcriptional level involving sirtuin 6, a member of the sirtuin family [46].

Some strengths and limitations of our study deserve additional comments. Among the strengths, our study is the first to consider simultaneous evaluation of NAD+ content and NFκB (p65) deacetylation in a murine model of T1D with a thorough biochemical characterization. Further, some limitations should also be acknowledged. First, circulating NAM concentrations were estimated and not directly determined in this experiment; however, genetically identical untreated T1D mice had significantly lower NAD+ in brains than NAM-treated, diabetic mice, thereby suggesting that, at least in part, NAD+ replenishment could be accounted for by increased NAM intake. Second, recent data suggest that NAM n-oxide, which is mainly produced by the intestinal microbiota from NAM, behaves as a positive regulator of sirtuin 1 of herpes simplex virus-induced brain inflammation and microglial [47]. As such, we cannot rule out the direct activation of the deacetylation machinery by this metabolite in our NAM-treated mice. Third, the potential involvement of a sirtuin-mediated mechanism was only indirectly revealed by the decreased relative abundance of the acetylated form of (p65) NFκB in the brains of NAM-treated, diabetic mice. Finally, the experimental design used in this study assessed the preventive but not the therapeutic effects of NAM on brain inflammation. Thus, further studies are warranted to confirm and expand the present observations.

## 5. Conclusions

NAM supplementation prevented brain inflammation and microglial activation in the brains of diabetic mice. These findings were directly related to concomitant increases in the brain content of NAD+ and concomitant deacetylation of the NFκB (p65) in the brains of diabetic mice treated with NAM. Our data might suggest increased rates of NAD+-dependent deacetylase in the brains of treated diabetic mice.

## Figures and Tables

**Table 1:** Gross and biochemical characteristics.

Variable	Non-T1D	T1D			*p*-Value
		Untreated	NAM LD	NAM HD	
*Gross*					
Final body weight (g)	29.16 ± 1.01	21.65 ± 1.26 *	21.69 ± 0.97 *	21.08 ± 0.67 *	<0.05
Liver weight (g)	1.22 ± 0.04	1.26 ± 0.07	1.17 ± 0.06	1.02 ± 0.08	0.2638
Kidneys’ weight (g)	0.38 ± 0.01	0.39 ± 0.01	0.38 ± 0.02	0.37 ± 0.02	0.4528
Brain weight (g)	0.40 ± 0.01	0.38 ± 0.00	0.39 ± 0.01	0.39 ± 0.01	0.9660
Food intake (g/day)	2.65 ± 0.20	7.11 ± 0.31 *	6.77 ± 0.92 *	7.52 ± 0.66 *	<0.05
Water consumption (mL/day)	4.73 ± 0.25	36.21 ± 2.41 *	37.67 ± 1.21 *	32.18 ± 1.57 *	<0.05
NAM intake (g/day/kg)	--	--	1.75 ± 0.10	3.73 ± 0.26 ‡	<0.05
*Plasma biochemistry*					
Glucose (mmol/L)	12.3 ± 0.6	40.7 ± 2.3 *	41.1 ± 2.3 *	41.7 ± 3.0 *	<0.05
Insulin (ng/mL)	0.65 ± 0.06	0.12 ± 0.03 *	0.26 ± 0.15 *	0.15 ± 0.02 *	<0.0001
AST (U/L)	272.0 ± 142.4	405.6 ± 139.1	364.6 ± 228.6	504.2 ± 211.9	0.8676
ALT (U/L)	60.15 ± 33.02	156.6 ± 41.92	45.53 ± 29.74	53.72 ± 6.95	0.1898
Total cholesterol (mmol/L)	3.06 ± 0.32	2.95 ± 0.15	3.96 ± 0.73	3.08 ± 0.23	0.1661
Triglycerides (mmol/L)	0.60 ± 0.13	1.61 ± 0.23 *	0.61 ± 0.10 †	0.93 ± 0.18	<0.01
*Brain NAD+*					
NAD+ (pmol/mg protein)	1.86 ± 0.45	1.01 ± 0.22 ^a^	3.67 ± 0.47 †	3.50 ± 0.16 †	<0.0001

Data are presented as mean (standard error) (n = 5–6 animals/group). ALT, alanine aminotransferase, AST, aspartate aminotransferase; NAM, nicotinamide; NAM LD, low-dose, NAM-treated, diabetic mice; NAM HD, high-dose, NAM-treated, diabetic mice; non-T1D, group of mice without T1D; T1D, group of mice with T1D; UN, untreated, diabetic mice. * *p*-value < 0.05 vs. non-T1D; † *p*-value < 0.05 vs. T1D; ‡ *p*-value < 0.05 vs. NAM LD T1D; ^a^ *p*-value = 0.08 vs. non-T1D.

## Data Availability

Proposals relating to the data access should be directed to the corresponding authors. To gain access, data requestors will need to sign a data access agreement.

## Supplementary Materials

The following supporting information can be downloaded at: https://www.mdpi.com/article/10.3390/nu15143083/s1, Figure S1: Negative controls for immunohistochemical staining. Figure S2. Effect of NAM on the gene expression of microglial markers in STZ-induced diabetic mice. Figure S3, western blot analysis.
