# Supplementary material for: Nicotinamide Prevents Diabetic Brain Inflammation via NAD+-Dependent Deacetylation Mechanisms
Source: Nutrients. 2023 Jul 9;15(14):3083. doi: 10.3390/nu15143083 (PMC10383777; doi:10.3390/nu15143083)
Supplement: Supplementary file 1 [file nutrients-15-03083-s001.zip › nutrients-2441339-supplementary.pdf]

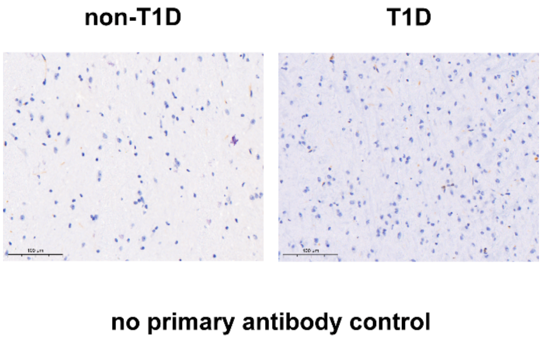

Figure S1. Negative controls for immunohistochemical staining. Representative images of negative controls for IHC staining shown in Figures 3-5, in brain tissue of non-diabetic and diabetic mice. Negative controls for the data shown in Figures 3-5 were performed for in different control blocks including brain tissue incubated with the secondary antibodies used for immunostaining (of which a cutout is shown). The scale bar shown in the images represents 100 μm.

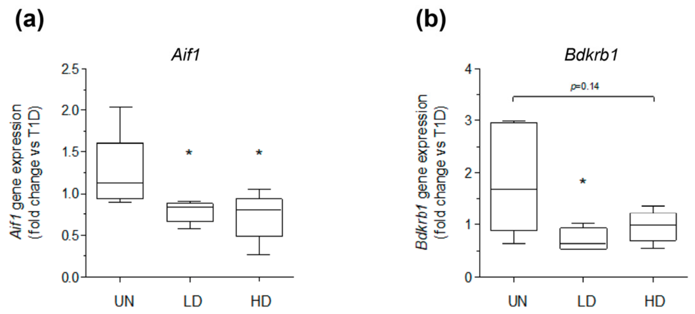

Figure S2. Effect of NAM on the gene expression of microglial markers in STZ-induced diabetic mice. (a) Relative gene expression of brain *Aif1*. (b) Relative gene expression of brain *Bdkrb1*. Abbreviations used: NAM LD, low-dose, NAM-treated mice; NAM HD, high-dose, NAM-treated mice. Data are expressed as the mean (standard deviation) of 4–5 mice/group. Statistically significant differences among groups for each variable were determined using a parametric ANOVA test followed by Tukey's posttest. Differences were considered significant when *p*-value <0.05. Specifically, * *p*-value <0.05 vs. untreated diabetic group.

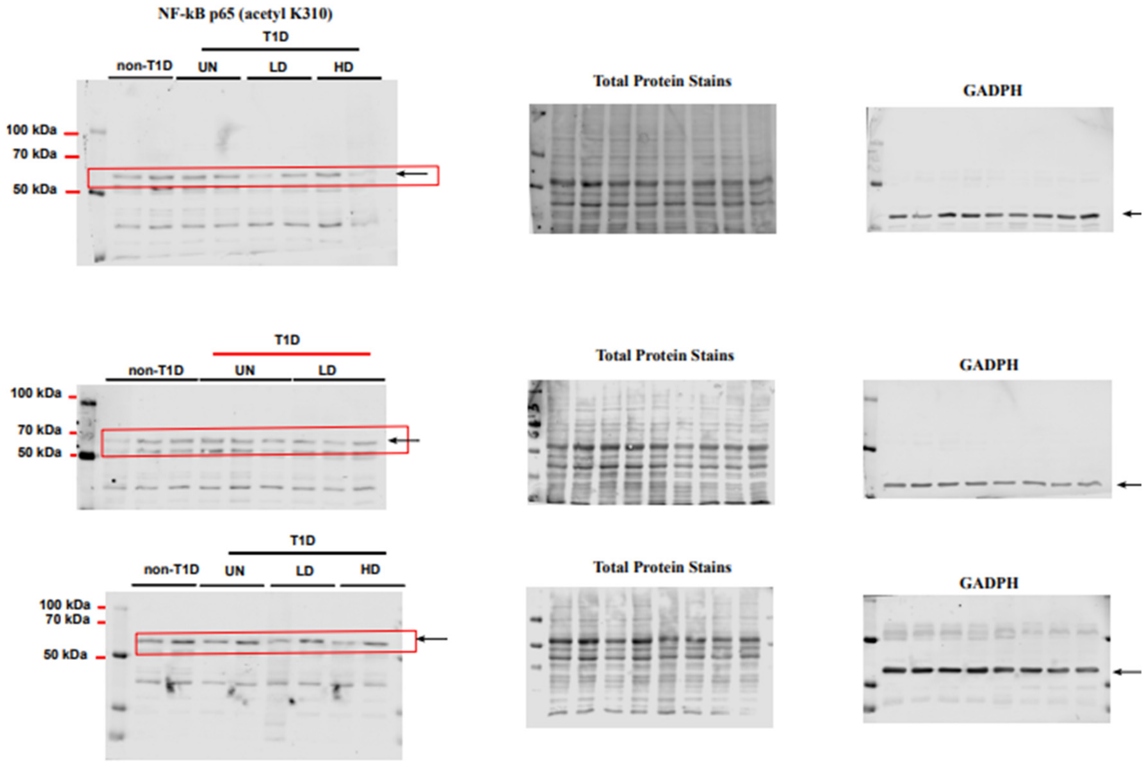

Figure.....

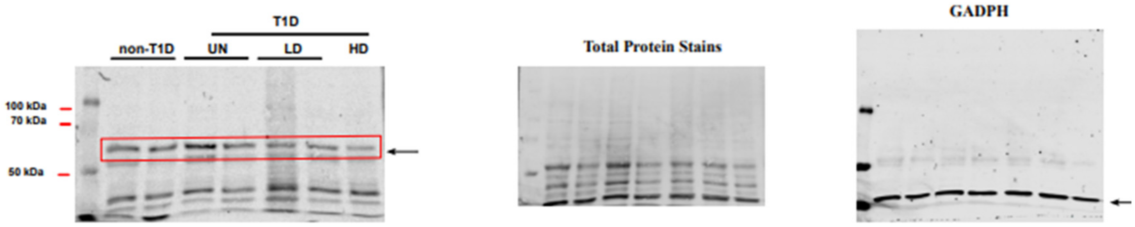

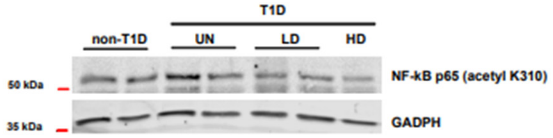

Figure S3. Effect of NAM on the protein abundance of deacetylated NFκB in brains of STZ-induced diabetic mice. Upper panels, set of western blots used for estimating the relative protein abundance of brain of acetylated NFκB. Bottom panels, western blot used to construct a representative western blot for acetylated NFκB in brain protein extracts shown in Figure 5.
